# Exploring the anti-HIV-1 reverse transcriptase, anti-inflammatory, anti-cancer activities and cytotoxicity of two fermented commercial herbal concoctions sold in Limpopo Province of South Africa

**DOI:** 10.1186/s12906-021-03321-2

**Published:** 2021-05-26

**Authors:** Matimba I. Ntlhamu, Ashwell R. Ndhlala, Peter Masoko

**Affiliations:** 1 Department of Biochemistry, Microbiology and Biotechnology, Faculty of Science and Agriculture, University of Limpopo, Private Bag X1106, Sovenga, Limpopo 0727 South Africa; 2 Green Biotechnologies Research Centre of Excellence, Faculty of Science and Agriculture, University of Limpopo, Private Bag X1106, Sovenga, Limpopo 0727 South Africa

**Keywords:** Anti-HIV-1 reverse transcriptase, Cytotoxicity, Anti-cancer, Fermented herbal concoctions

## Abstract

**Background and objectives:**

The use of herbal concoctions is very popular in South Africa, including Limpopo Province. The herbal concoctions are claimed to be capable of treating numerous illnesses such as ulcers, cancer, HIV/AIDS, diabetes, certain STDs, blood cleansing to mention but a few. The focus of this study was to evaluate the anti-HIV 1 reverse transcriptase, anti-inflammatory and anti-cancerous activities as well as cytotoxic effects of 2 fermented herbal concoctions used for the treatment of the related ailments in Limpopo province of South Africa.

**Method:**

Two fermented herbal concoctions obtained from a herbalist in Polokwane were extracted with 80% acetone. The anti-HIV activity of the herbal concoctions was determined using the anti-HIV reverse transcriptase assay. The anti-cancer and cytotoxic effects of the herbal concoctions were evaluated using cancerous Human Colon (HT-29) cells and the normal human Hepatoma cells (C3A) respectively.

**Results:**

Notable anti-HIV reverse transcriptase activity was observed from the 80% acetone fraction of herbal concoction 1 (IC_50_ 38.031 μg/mL) which exhibited better activity than the positive control Lamivudine (IC_50_ 40.90 μg/mL). There was variation in the anti-inflammation activity as determined by the sPL2, 15-LOX and COX enzyme assays. The only concerning matter was the high COX-1 activity in some of the extracts, which is not desirable due to the mucosal protection action of COX-1 enzyme. The herbal concoctions did not exhibit cytotoxic effects on normal human cells, however, toxicity against cancerous cells was observed.

**Conclusion:**

The herbal concoctions displayed some considerable pharmacological effects against various ailments as claimed by the herbalist. More work to ascertain the toxicity of both concoctions against cancerous cells need to be followed as this could lead to the discovery of anticancer drugs.

## Background

Since ancient times, plants have been used as medicine throughout the world [1]. In South Africa, there is great cultural diversity and several ethnic groups, which results in the massive use of medicinal plants throughout the provinces [2]. Each cultural group in South Africa has different medical solutions for the prevention and curing of the same disease [3]. Hence, several ethnic groups use traditional knowledge to cure various infectious diseases caused by parasites, bacteria and viruses in addition to the treatment of poisonings caused by snakes and scorpions as well as skin diseases, inflammations, bronchial conditions, fever and pains [4–6].

The World Health Organization (WHO) has estimated that 80% of the world’s population use traditional medicine for their health care needs and it commonly requires the use of herbal extracts and their active components. There are approximately 20,000 herbal plants in the world used for medicinal purposes [7]. In South Africa, there are 30,000 species of higher plants and there are approximately 350 species which are traded as medicinal plants [8].

Medicinal plants used traditionally to treat infectious diseases seem to be an abundant source of new bioactive secondary metabolites. Therefore, more effective antimicrobial agents with novel modes of action must be discovered and developed. The World Health Organization (WHO) recommended that traditional healers be included in national responses to HIV/AIDS [9]. As early as 1989, WHO had appealed to the need to evaluate ethnomedicine for the management of HIV/AIDS. In this regard, several studies have reported anti-HIV potential of medicinal plants and their derivatives.

Traditionally, plant medicines are often used as extracts, where some of these extracts are a mixture of different plant parts, commonly referred to as herbal concoctions. Concoctions could also be some simple and common homemade remedies made to treat minor illnesses as well as complex remedies used to treat major and life-threatening illnesses [10] (Cano and Volpato, 2004). In South Africa, concoctions are used as traditional medicines as it is claimed that they possess disease healing properties such as HIV/AIDS and other related infections [11]. Plant parts commonly used for the preparation of herbal concoctions include stems, leaves, roots, bark, etc. [12]. Different methods are used to prepare herbal concoctions, starting from simple brewing processes to more complex techniques that use alcohol and other organic solvents to extract plant compounds [13]. Fermentation is a microorganism driven process which yields high value product from raw or low-grade substrates. Hence some herbal products are fermented in order to break down or convert the undesirable substrates into compatible components under the action of microbial enzymes, thereby improving the substrate properties via the production and enrichment of bioactive compounds. In addition, fermentation improves the nutrient values of foods and breaks them down into more easily catabolisable forms [14]. The advantage of concoctions lies in their composition as rich mixtures of different plants. Furthermore, the herbal concoctions possess synergistic and additive pharmacological effects that result from mixing different plants [15]. Herbal products of cooperate manufacturers have standard formulae; they are fully labelled according to how they are used and are stored in appropriate conditions whereas those from herbalists are just prepared and stored like the ones at *muthi* shop owners and street vendors. Hence the poor regulations of these concoctions pose a threat to customer’s health.

The HIV epidemic continues to be a major global public health issue. In the year 2017, there were 25.7 million people living with HIV/AIDS in Sub-Saharan Africa, accounting for two-thirds of new HIV infections globally [16]. The Joint United Nations Programme on HIV/AIDS (UNAIDS) FastTrack strategy aims to increase the HIV response in low and middle-income countries to end the epidemic by 2030 [17]. A study has previously shown that most people living with HIV/AIDS are susceptible to fungal and bacterial opportunistic infections that result from immunosuppression [18]. South African health researchers and clinicians have been actively involved in human immunodeficiency virus (HIV) research for more than three decades now, with prevention and treatment as the main focus [19].

Moreover, the use of medicinal plants is associated with irritation of the gastrointestinal tract, destruction of red blood cells and damage of the heart and kidney [20]. Therefore, this necessitates the need for toxicity evaluation of herbal concoctions used in ethnopharmacology. In vitro*,* toxicological studies use a wide number of assays to determine cytotoxicity that comes from the exposure of chemical substances [21].

It has been reported by the World Health Organisation (WHO), that cancer is one of the leading causes of morbidity and mortality worldwide [22]. Cancer, according to the National Cancer Institute (NCI) of the United States of America, is described as a collection of associated diseases. It results from uncontrollable cell division which leads to deregulation between cell death and cell proliferation. This leads to formation of tumours, as cells that should have died did not receive the signal to do so [23]. These tumours can either be malignant or benign [24]. Malignant tumours contain cells that are capable of detaching, migrating and forming secondary tumours in other parts of the body. In contrast, benign tumours have cells that proliferate and remain at the site of origin [25].

A global increase of 1% in cancer-related deaths was reported between the years 2011 and 2013. These cases are expected to increase by 46% over the next 13 years [26]. Hence due to the high cancer mortality rate, the development of drug resistance, as well as undesirable side effects, there is a pressing need to search for and/or develop new anti-cancer drugs [27]. There exist several pervasive developments in methods for synthesis of cancer therapeutic drugs in the pharmaceutical industry, however, medicinal plants still represent important sources of new molecular identities. This is because plants can synthesise and produce components that are burdensome to obtain through chemical synthesis and this makes them an important source for the development of new anticancer drugs that would perhaps selectively kill cancerous cells [28].

The National Cancer Institute-USA has screened roughly 35,000 plant species for potential anticancer activities. Amongst these, approximately 3000 plant species were found to have anticancer activity [29]. There is still a large reservoir of bioactive compounds, however, only a few have been examined thus far and continue to be a principal potential source of anticancer agents [30].

In the Limpopo Province (Mankweng), there is a strong rise of herbal medicine production and trading by herbalists. The herbalists primarily sell herbal concoctions which apart from being prescribed for the treatment of HIV, ulcers and cancer, they are claimed to have aphrodisiac, immune-boosting and blood cleansing effects, however, the efficacy and safety of the concoctions have not been validated. It is therefore the objective of this study to validate the anti-HIV properties of the fermented herbal concoctions and the potentially toxic effects that may arise from the consumption of the concoctions and will be evaluated through the determination of cell viability after 24-h exposure.

## Methods

### Concoction preparation

Two commercially available fermented herbal concoctions were supplied by an herbalist (Mr. MS Mathebula) based at the University of Limpopo. Once procured, the fermented herbal concoctions were separately subjected to a stream of cold air to drive out any alcohol by products before freeze drying. The dried samples were partitioned into two from each herbal product, the crude extract while the other portion was extracted with 80% acetone. The crude extracts were reconstituted with water and the other with 80% aqueous ethanol. The former considered as the crude and the latter, regarded as a sub fraction. Table 1 list the plant material used to produce the herbal concoctions. The collection of plant materials by the herbalist is done in compliance with the guidelines set in the South African Bureau of Standards on collection and harvesting of Medicinal plants (SANS ARP 029:2013).

**Table 1:** Plant species used as ingredients of two fermented commercial herbal concoctions

Herbal Mixture	Plant composition		Plant part
	Family name	Scientific name	
1	Geraniaceae	*Monsonia angustifolia* E.Mey. ex A.Rich.	Leaf
	Oleaceae	*Olea europaea* L.	Seed
2	Fabeae	*Lens esculentum* Mill.	Leaf + Stem
	Moringaceae	*Moringa oleifera* Lam.	leaf
	Vitaceae	*Vitis vinifera* L.	Fruit pulp

### HIV-1 reverse transcriptase (RT) inhibitory bioassay

The effect of the herbal preparations on reverse transcription was evaluated using a non-radioactive HIV-RT colorimetric ELISA kit obtained from Roche Diagnostics, Germany and detailed by Ndhlala et al. [31] with modifications [32]. The protocol supplied together with the kit was followed, under nuclease-free conditions. The reverse transcriptase colorimetric assay takes advantage of the ability of RT to synthesize DNA, starting from the template/primer hybrid poly (A) × oligo (dT)15. The kit avoids the use of [^3^H]- or [^32^P]-labelled nucleotides which are used for the other classical RT assays. In place of radio-labelled nucleotides, digoxigenin- and biotin-labelled nucleotides are incorporated into one and the same DNA molecule, which is freshly synthesised by the RT. The detection and quantification of synthesized DNA as a parameter for RT activity is followed in a sandwich ELISA protocol: Biotin-labelled DNA freshly synthesised by the RT binds to the surface of microtiter plate modules (MPM) with wells that were precoated with streptavidin. In the next step, an antibody to digoxigenin, conjugated to peroxidase (anti-DIG-POD), binds to the digoxigenin-labelled DNA. In the final step, the peroxidase substrate ABTS (2, 2′-Azinobis [3-ethylbenzothiazoline-6-sulfonic acid]- diammonium salt) is added. The peroxidase enzyme catalyses the cleavage of the substrate, producing a coloured reaction product which is measured spectrophotometrically.

The following solutions provided with the kit were prepared according to the manufacturer; Solution 1, HIV-1 reverse transcriptase (final concentration 2 ng/μL, corresponding to 10 mU/μL) stored at − 70 °C. Solution 2, incubation buffer. Solution 3, reaction mixture containing poly (A) x oligo (dT)15 (46 mM Tris-HCl, 266 mM potassium chloride, 27.5 mM magnesium chloride, 9.2 mM DDT, 10 μM dUTP/dTTP, template/primer hybrid, 750 mA260 nm/mL). Solution 4, lysis buffer. Solution 5, anti-digoxigenin-peroxidase (anti-DIG-POD) (200 mU/mL). Solution 6, washing buffer and solution 7, ABTS substrate solution. In sterile Eppendorf tubes, 20 μL of resuspended herbal preparations (with final assay concentrations of 0.25, 2.5, 25, 250, 2500 μg/mL) or controls were mixed with 20 μL of recombinant HIV-1-RT (4 ng in lysis buffer) and 20 μL reaction mixture (solution 3) and the tubes were incubated for 1 h at 37 °C. After the 1 h incubation period, the contents of the tubes (60 μL) were transferred into MPM wells. The MPM was covered with foil and incubated for 1 h at 37 °C after which the contents were removed from the MPM wells completely. The wells were rinsed 5 times with 250 μL of washing buffer (solution 6) per well for 30 s, with the washing buffer being removed carefully after each wash. After the wash, 200 μL of anti-DIG-POD (solution 5) was added to each well and the MPM was re-covered with foil and incubated for 1 h at 37 °C. After the incubation period, the solution was removed completely from the MPM wells. The MPM wells were rinsed 5 times with 250 μL of washing buffer (solution 6) per well for 30 s, the washing buffer was removed carefully after each wash. After washing, 200 μL of ABTS substrate solution (solution 7) was added to each well and the MPM was incubated at room temperature for 5 min (a green colour appeared in the wells). The absorbance of the reaction mixture was then measured at 405 nm (reference wavelength: 490 nm) using a microplate reader (Opsys MR™, Dynex Technologies Inc.). Percentage of inhibition was calculated by comparing the absorbance of the sample to the negative control using the equation below:

$$ \mathrm{HIV}-1\ \mathrm{RT}\ \mathrm{inhibition}\left(\%\right)=\left\{1-\left(\frac{{\mathrm{Abs}}_{405\ \mathrm{nm}}\ \mathrm{Sample}\ }{{\mathrm{Abs}}_{405\ \mathrm{nm}}\ \mathrm{Neg}\ \mathrm{Control}}\right)\right\}\times 100 $$

where Abs_405nm_ Sample is the absorbance of the reaction with herbal preparations or positive control at 405 nm and Abs_405nm_ Neg Control is the absorbance of reaction with water instead of the sample at 405 nm.

Three tubes containing water instead of the sample were used as negative controls. Combivir® (GlaxoSmithKline) [lamivudine (1.0 mg/mL) + zidovudine (2.0 mg/mL)] and Kaletra® (Abbott) [lopinavir (8.9 mg/mL) + ritonavir (2.2 mg/mL)] were used as positive controls.

Results were presented as means duplicates ± standard deviations of 2 independent experiments; each experiment was done in duplicate. The IC_50_ values of herbal preparations were calculated using Graph Pad Prism (version 5.0).

### Anti-inflammatory assay

#### Secretory phospholipase (sPLA2) inhibitory activity assay

The anti-inflammatory activity of the herbal concoctions was evaluated by examining the inhibition of the human sPLA2 enzyme using the sPLA2 (Type V) inhibitor screening assay kit (Cayman Chemical, Ann Arbor, MI) as described by George et al., [33] with modifications [34]. The assay measures free 5-thio-2-nitrobenzoic acid (TNBA) following hydrolysis of diheptanoylthio-phosphatidylcholine (PC) by sPLA2. Briefly, 10 μL of the sPLA2 was dissolved in assay buffer solution (25 mM Tris–HCl, 10 mM CaCl_2_,100 mM KCl, 0.3 mM Triton X-100, pH 7.5) and 10 μL of concoction extract at concentrations of 50, 25, 12.5 and 6.25 μg/mL were added into a 96-well microtiter plate. The reaction was initiated by adding 200 μL of a substrate solution (diheptanoyl thio-PC, 1.66 mM), covered with aluminium foil and incubated at 25 °C for 15 min. After incubation, 10 μL of DTNB (5,5′-dithio-bis-2-nitrobenzoic acid; 10 mM, 0.4 M Tris–HCl, pH 8.0) was added into each well. The reaction mixture containing the assay buffer and solvent served as the blank and mixture with sPLA2 enzyme and solvent served as the 100% initial activity (IA). Thioetheramide-PC served as the positive control. The hydrolysed diheptanoyl thio-PC was measured at 420 nm using a microplate reader (Optic Iveymen®System, Model 2100-C). The percentage inhibition was calculated using the formula below. The percentage inhibition was plotted against plant extract concentration and the IC_50_ was determined from the normalised logarithmic regression curve.

$$ {\mathrm{sPLA}}_2\%\mathrm{Inhibition}=\left[\left(100\%\mathrm{IA}-\mathrm{Inhibition}\right)/100\%\mathrm{IA}\times 100\right] $$

where IA is inhibition absorbance.

#### Lipoxygenase (15-LOX) inhibitory activity assay

The anti-inflammatory activity of the herbal concoctions was evaluated by examining the inhibition of the 15-LOX enzyme using the LOX inhibitor screening assay kit (Cayman Chemical, Ann Arbor, MI) as described by Boudjou et al. [35] with modifications [34]. The assay measures hydroperoxides produced in the lipoxygenation reaction using purified 15-LOX. Briefly, 90 μL of 15-LOX was dissolved in assay buffer solution (0.1 M Tris–HCl, pH 7.4) and 10 μL concoctions extract at a concentration of (50, 25, 12.5 and 6.25 μg/mL) was added into each well of a 96-well microtiter plate. The plate was incubated at 25 °C for 5 min. After incubation, the reaction was initiated by adding 10 μL substrate solution of arachidonic acid (1 mM) and mixed on a shaker for 10 min. The reaction was stopped by adding 100 μL of the chromogen into each well. The reaction mixture containing the assay buffer served as the blank and mixture with 15-LOX and solvent served as the 100% IA. Nordihydroguairetic acid (NDGA) served as a positive control. The absorbance was measured at 490 nm using a microplate reader (Optic Iveymen® System, Model 2100-C). The percentage inhibition was calculated using the formula below. The percentage inhibition was plotted against concoction extract concentration and the IC_50_ determined from the normalised logarithmic regression curve.

$$ \mathrm{LOX}\%\mathrm{Inhibition}=\left[\left(100\%\mathrm{IA}-\mathrm{Inhibition}\right)/100\%\mathrm{IA}\times 100\right] $$

where IA is inhibition absorbance.

#### Cyclooxygenase (COX-1 and COX-2) inhibitor screening assay

The anti-inflammatory activity of the two herbal concoctions was evaluated by examining the inhibition of the ovine COX-1 and human COX-2 enzyme using the COX inhibitor screening assay kit (Cayman Chemical, Ann Arbor, MI) as described by Boudjou et al. [35] with modifications [34]. The assay measures the peroxidase activity of ovine COX-1 and human COX-2, by monitoring the appearance of oxidised N, N, N′, N′-tetramethyl-p-phenylenediamine (TMPD). Briefly, 150 μL assay buffer (0.1 MTris-HCl, pH 8), 10 μL heme and 10 μL ovine COX-1 enzyme was added to each well of the 96-well microtiter plate. The same procedure was repeated with the human COX-2 enzyme. A volume of 10 μL concoction extract at a concentration of 50, 25, 12.5 and 6.25 μg/mL was added to each well. The plate was carefully mixed by shaking for 30 s and followed by incubation at 25 °C for 5 min. After incubation, 20 μL of TMPD was added to each well of the 96-well microtiter plate and the reaction was initiated by the addition of 20 μL arachidonic acid. The plates were further incubated at 25 °C for 5 min. The reaction mixture containing assay buffer and heme served as the blank and a mixture with either COX-1 or COX-2 enzyme, assay buffer and heme served as the 100% IA. Indomethacin served as a positive control. The absorbance of oxidised TMPD was read at 490 nm using a microplate reader (Optic Iveymen® System, Model 2100-C). The percentage inhibition was calculated using the formula below. The percentage inhibition was plotted against concoction extract concentration and the IC_50_ was determined from the normalised logarithmic regression curve. Data were expressed as means of duplicates ± standard deviations.

$$ \mathrm{COX}\%\mathrm{Inhibition}=\left[\left(100\%\mathrm{IA}-\mathrm{Inhibitor}\right)/100\%\mathrm{IA}\times 100\right] $$

where IA is inhibition absorbance.

### Cytotoxicity and anti-cancer assay

To determine the toxicological outcomes of the consumption of the concoctions, their cytotoxic effect on normal human hepatoma cell lines (C3A). The anti-cancer effect of the herbal concoctions was evaluated on human colon (HT-29) cancer cells. The 3-(4,5-dimethylthiazol-2-yl)-2,5-diphenyltetrazolium bromide (MTT) colorimetric assay described by Mosmann [36] was performed with modifications [34]. Human colon (HT-29) cancer cells and human hepatoma cell lines (C3A) (ATCC® HTB-38) (Biosafety level 2) were purchased from the American Type Culture Collection (ATCC). The cells were maintained in a flask with Dulbecco’s Modified Eagle’s Medium (DMEM), (Whitehead Scientific) supplemented with 10% v/v foetal bovine serum (FBS) (Adcock-Ingram) in a cell culture incubator at 37 °C in humidified air containing 5% carbon dioxide (CO_2_). Cells (1 × 105 cells/mL) were seeded in a 96-well plate and allowed to attach overnight in a cell culture incubator. Cells were then treated with various concentrations (100–900 μg/mL) of different herbal concoctions for 24 h. Following treatment, 20 μL of 5 mg/mL MTT was added and the cells were further incubated for 4 h. The MTT solution was then removed and 200 μL of dimethyl sulfoxide (DMSO) was added to dissolve the MTT formazan crystals. Purple formazan crystals are formed when MTT is reduced by metabolically active cells. Thus, the number of formed formazan products produced indicates the number of viable cells. For quantification, the absorbance was measured at 560 nm using the GloMax®-Multi+Detection System microtitre plate reader (Promega).

### Statistical analysis

The data were expressed as mean ± standard deviation (S.D). Statistically significant differences between the untreated control and treatments were determined using the GraphPad Software (Version 5, San Diego, CA). Samples were treated to one-way ANOVA, followed by Dunnett’s comparison test. Differences between means of untreated, treated cells were considered significant at *p* ≤ 0.05 (*) and highly significant at *p* ≤ 0.01 (**).

## Results and discussion

The human immune-deficiency virus (HIV)-1 reverse transcriptase is a very important enzyme in the HIV life-cycle, it transcribes its ribonucleic acid (RNA) code to synthesise a viral deoxyribonucleic acid (DNA) that invades other cells [37]. Therefore, this makes it a crucial target towards the screening and development of antiretroviral drugs against HIV. The anti-HIV potential of the herbal concoctions was evaluated by determining their capability to inhibit the HIV-1 reverse transcriptase (RT) activity. The concentration of an inhibitor that can produce half (50%) maximal activity was denoted as IC_50_. Therefore, the smaller the concentration (IC_50_) of a drug required to inhibit HIV-RT whilst exhibiting minimum or no toxicities can be considered for pharmaceutical development. Four levels of activity were defined as follows: activity below 25 μg/mL being considered high activity, 25–50 μg/mL good activity, 50–100 μg/mL moderate activity and above 100 μg/mL low activity. These levels of activity were also used for defining activities in the other assays including the sPLA2, LOX and COX enzyme inhibitor activity assays in this article.

The results demonstrated that only the sub fraction of herbal concoction 1 showed good activity (38.031 μg/mL). This activity was higher than the positive control Lamivudine (40.90 μg/mL) (Table 2). This activity of the sub fraction of herbal concoction 1 suggested that it may be able to inhibit the early phases of the HIV-1 replicative cycle that is mediated by the HIV-RT. Ndhlala et al. [31] reported high HIV-RT inhibitory effects of aqueous herbal concoctions sold in KwaZulu-Natal (South Africa). Moreover, Matotoka et al. [32] reported the in vitro inhibition of HIV-1 reverse transcriptase of some herbal concoctions sold in the Limpopo Province. The rest of the other samples as obtained from the two fermented herbal concoctions exhibited variable activity against anti-HIV (Table 2) with crude sample of herbal concoction 1, crude and sub fraction (80% acetone) of herbal concoction 2 exhibiting moderate activity with IC_50_ values ranging from 56.112 to 88.323 μg/mL. The differences in activity of the concoctions may stem from the manner of preparation procedures taken to prepare the herbal constituents.

**Table 2:** HIV-1 reverse transcriptase activity and anti-inflammatory properties of some fermented herbal concoctions sold in Limpopo

Sample	HIV-RT 1 (IC_50_ μg/mL)	sPLA2 (IC_50_ μg/mL)	15-LOX (IC_50_ μg/mL)	COX-1 (IC_50_ μg/mL).	COX-2 (IC_50_ μg/mL).
C1 (Crude)	64.4 ± 2.1^b^	70.4 ± 0.1^a^	88.2 ± 0.2^a^	2.11 ± 0.01^d^	18.00 ± 0.01^d^
C1 (80% Acetone)	39.5 ± 1.6^de^	25.1 ± 1.3^b^	67.2 ± 0.6^d^	5.43 ± 0.05^b^	27.60 ± 0.02^b^
C2 (Crude)	86.1 ± 1.8^a^	15.5 ± 0.6^c^	56.1 ± 0.3^c^	3.67 ± 0.02^c^	22.10 ± 0.01^c^
C2 (80% Acetone)	54.5 ± 1.5^c^	15.3 ± 0.5^c^	42.3 ± 0.1^d^	6.72 ± 0.01^a^	34.10 ± 0.01^a^
Lamivudine	41.0 ± 0.2^d^				
Zidovudine	24.9 ± 0.6^g^				
Lopinavir	33.8 ± 0.5^f^				
Ritonavir	36.5 ± 0.3^ef^				
Thioetheramide-PC		1.52 ± 0.1^d^			
Nordihydroguairetic acid			10.8 ± 0.1^e^		
Indomethacin				0.59 ± 0.01^e^	8.70 ± 0.10^e^

C1- Concoction 1; C2- Concoction 2. Data are represented as means duplicates ± standard deviations. Different letters in columns are significantly different

The IC_50_ results for the sPLA2 inhibitor activity assay are represented in Table 2. The results indicated that the tested extracts had IC_50_ values lower than the positive control. The inhibition of sPLA2 activity can be a beneficial towards the progression of HIV and cancer because of lower levels of free arachidonic acid. Free arachidonic acids are metabolised into eicosanoids by LOX and COX enzymes, resulting in the proliferation of acute inflammation [38] which may accelerate the progression of many diseases and conditions including HIV and cancer. Both crude and sub fraction of herbal concoction 2 expressed notable inhibitory activity against sPLA2 with IC_50_ values of the two being closely around 15 μg/mL when compared to the samples derived from the reconstitutes of herbal concoction 1. The lowest activity against sPLA2 was recorded for crude concoction 1 with an IC_50_ value around 70 μg/mL (Table 2).

The inhibition of 15-LOX was used to determine the anti-inflammatory activity of the extracts with the results expressed as IC_50_ values (Table 2). The free arachidonic acid released by the activity of sPLA2 is metabolised by 15-LOX to form HETE which results in the proliferation of atherosclerotic plaque formation. The results indicated that the tested concoctions had IC_50_ values ranging from 88 to 42 μg/mL (Table 2). The sub fraction of herbal concoction 2 expressed the highest inhibitory activity against 15-LOX with an IC_50_ value around 42 μg/mL.

The inhibition of COX-1 and COX-2 was also used to indicate the anti-inflammatory activity of the extracts with the results expressed as IC_50_ values (Table 2). The tested extracts exhibited high activities even though they were low when compared to the positive control against COX-1 activity. Extracts with high inhibitory activity against COX-1 are a concern due to reported beneficial effects associated with COX-1 activity compared to its inhibition [39]. In this case, all the samples exhibited high inhibiting activity against COX-1 with IC_50_ values of 2.11 and 6.73 μg/mL. The COX-1 enzyme initiates the production of beneficial prostaglandins responsible for the maintenance and protection of the intestinal mucosal layer [40]. In the absence of COX-1, the mucosal layer is exposed and could result in ulceration. Therefore, there presence a challenge and negativity in use of these herbal concoctions due to persistent ulcerations associated with the inhibition of COX-1 activity.

However, the lower activities against sPLA2 encountered, necessitate exploration of other mechanisms by which the extracts may induce their inhibitory effect against the enzyme.

Most phytochemicals are foreign to the human body, therefore, the use of these bioactive compounds can be accompanied by detrimental toxicities and/or adverse effects that may impact the morbidity and mortality rate of the global populace. In this study, a colorimetric cell viability assay was used to determine the toxicological effects of herbal concoctions, where the tetrazolium salt 3-[4,5-dimethylthiazol-2-yl]-2,5-diphenyltetrazolium bromide (MTT) was used as an indicator. This salt is converted to an insoluble purple formazan by metabolically active cells. The tetrazolium ring is cleaved by succinate dehydrogenase found in the mitochondria. Due to the impermeability of the resulting formazan, this purple product is collected inside healthy cells. Upon resuspension, the absorbance of the purple colour displays the number of viable cells that are estimated [41]. This method is easy to use, safe, has a high reproducibility, and is widely used to determine both cell viability and cytotoxicity tests [42]. Moreover, In vitro cytotoxicity and/or cell viability assays have some advantages, such as speed, reduced cost and potential for automation, and tests using human cells may be more relevant than some In vivo animal tests [42, 43].

**Fig. 1:**
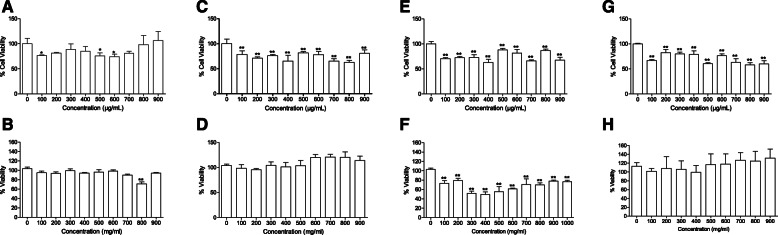
**a-d** Effects of crude herbal concoction 1 [**a (**HT-29) and **b (**C3A)], and 80% acetone herbal concoction 1 [**c (**HT-29) and **d** (C3A)] extracts on viability of human colon (HT-29) cancer cells and human hepatoma cell lines (C3A). Cells were incubated with the extract at the indicated concentrations for 24 h. The effect of the extract was determined using the MTT assay. Each data point represents the mean ± S.D. ** *p* ≤ 0.01, indicate significant differences to the untreated control. Effects of crude herbal concoction 2 [**e (**HT-29) and **f (C3A**)], and 80% acetone herbal concoction 2 [**g (**HT-29) and **h (C3A**)] extracts on viability of human colon (HT-29) cancer cells and human hepatoma cell lines (C3A)**.** Cells were incubated with the extract at the indicated concentrations for 24 h. Each data point represents the mean ± S.D. ** *p* ≤ 0.01, indicate significant differences to the untreated control

Cytotoxicity and anti-cancer outcomes of the consumption of the concoctions, were tested using normal human hepatoma cell lines (C3A) and human colon (HT-29) cancer cells. The percentage cytotoxicity and anti-cancer cell viability of herbal concoctions tested is represented in Fig. 1. A general observation after the 24 h incubation of the treated cell lines was that the viability of the cells was not entirely dependent on the increase and decrease of the concentrations and it was observed that there was no high consistency of the increase and decrease in cell viability percentages for both H-T29 and C3A cells. Overall, both concoction 1 and concoction 2 appeared to possess some very slight toxicity against cells in their crude form but the toxicity was very less in C3A. Both concoctions had potential anti-cancer properties at the concentrations of 700 and 800 μg/ml. It can be concluded that crude concoction 1 is not toxic against normal C3A cells at lower concentrations (Fig. 1). All C3A cells treated with sub fractions of concoction 2 were displaying very high percentages of viability at all the concentrations. Crude concoction 1 proved to be more toxic at higher concentrations (800 μg/ml) displaying a low percentage of cell viabilities against C3A (Fig. 1). However, this was not the case for HT-29 cancer cells (Fig. 1) which were not affected at of the same concentration. The crude concoction 2 proved to be highly toxic at 300 and 400 μg/ml concentrations displaying the lowest percentages of cell viabilities against HT-29 cancer cells as compared to its other concentrations. Moreover, it should be noted that both C3A and HT-29 have almost similar results of cell toxicity levels at the concentrations of 300 and 400 μg/ml. As the concentrations increased further, the crude concoction 2 proved to be more toxic against normal C3A cells than HT-29 cancer cells. Acute toxicity seems likely on crude concoction 2. Hence, all concoctions proved to possess activity against HT-29 cancerous cells as toxicity was found to be common in all tested concoctions. The anti-cancer activity of the concoctions does not result in toxicity against normal human cells; hence more work is needed to understand the mechanisms of action involved in cell selection.

The major chemical composition of the plant constituency of the two herbal mixtures are presented in Table 3. The main class of compounds gathered from literature are the phenolic compounds which in many studies are have attributed to anti-inflammation, anticancer and a lot other functionalities. Chief amongst these phenolic compounds are quercetin, kaempferol, ellagic acid, gallic acid and ferulic acid which all are known antioxidants [71, 72, 75]. Herbal mixture number two contains plant species which has rutin, *Vitis vinifera* L. and *Moringa oleifera* Lam., which is an important plant metabolite with vast pharmacological properties including anti-bacterial, anti-inflammatory, analgesic, anti-radiation, antioxidant and anti-myocardial hypoxia activity [73]. Some of these properties have been cited as the prime use of the herbal mixture and activity have been confirmed in the tests done in this and other studies.

**Table 3:** Major chemical constituents in the plant species used as ingredients of two fermented commercial herbal concoctions

Herbal Mixture	Scientific name	Chemical composition	Activity	Reference
1	*Monsonia angustifolia* E.Mey. ex A.Rich.	Triterpenoids (ursolic acid and oleanolic acid)	Anti-inflammatory and antitumour activities	[12]
		Cardiac glycosides	Anti-inflammatory	[12]
		Steroids	Anti-inflammatory and antitumour activities	[12, 44]
		Tannins	Antitumor activity, antiviral activity, and inhibition of active oxygen, such as inhibition of lipid peroxidation and lipoxygenase, xanthine oxidase, and monoamine oxidase	[12, 45]
		Justicidin A [9-(1′,3′-benzodioxol-5′-yl)-4,6,7-trimethoxynaphtho[2,3-c]furan-1(3H)-one]	Antifungal, antiviral and antibacterial activities	[46]
		5-Methoxyjusticidin A [9-(1′,3′-benzodioxol-5′-yl)-4,5,6,7-tetramethoxynaphtho[2,3-c]furan-1(3H)-one]	Anticholinesterase	[46]
		Chinensinaphthol [9-(3′,4′-dimethoxyphenyl)-4-hydroxy-6,7-methylenedioxynaphtho[2,3-c]furan-1(3H)-one]		[46]
		Retrochinensinaphthol methyl ether [4-(3′,4′-dimethoxyphenyl)-9-methoxy-6,7-methylenedioxynaphtho[2,3-c]furan-1(3H)-one]	Anthelmintic activity	[46, 47]
		Suchilactone [3-(1′,3′-benzodioxol-5′-ylmethylene)-4-(3″,4″-dimethoxybenzyl)dihydrofuran-2(5H)-one]	Antioxidant activity	[46]
		Secoisolariresinol	Antioxidant activity	[48]
		Matairesinol	Anticancer, Antidiabetes, and protective against heart diseases	[48]
		Podophyllotoxin	Antineoplastic and antiviral properties	[48, 49]
		Desoxypodophyllotoxin	Antitumor and anti-inflammatory activities	[48, 50]
	*Olea europaea* L.	Oleuropein	Antioxidant, antiinflammatory, anti-atherogenic, anti-cancer, antimicrobial and antiviral activity	[51–53]
		Caffeic acid-C-hexoside	Antioxidant, antiinflammatory	[54, 55]
		Coumaric acid	Antioxidant, anti-inflammatory activity	[54, 55]
		Citric acid	Antioxidant activity	[54]
		Hydroxytyrosol	Anti-inflammatory, anti-tumor, antiviral, antibacterial and antifungal properties	[54, 56]
		Verbascoside	Antioxidant, anti-inflammatory and antineoplastic properties	[54, 57]
		elenolic acid	Antioxidant, antiinflammatory, anti-atherogenic, anti-cancer, antimicrobial and antiviral activity	[53, 54]
		ligstroside	Antioxidant activity	[54]
		Gallic acid	Antioxidant, anti-inflammatory, and antineoplastic properties	[52, 58]
		Rutin	Anti-bacterial, anti-inflammatory, analgesic, anti-radiation, antioxidant, anti-myocardial hypoxia activity	[51, 52, 58]
		Apigenin-7-Glucoside	Anti-inflammatory, antioxidant activity	[51, 52, 58]
		Quercetin	Anti-inflammatory, antioxidant activity	[58]
		Ferulic acid	Anti-inflammatory, antimicrobial, anticancer, anti-arrhythmic, and antithrombotic activity, antidiabetic effects and immunostimulant properties	[58, 59]
		Kaempferol	Antioxidant, anti-inflammatory, antimicrobial, anticancer, cardioprotective, neuroprotective, antidiabetic, anti-osteoporotic, antiestrogenic activities	[52, 60]
		Luteolin	Antioxidant activity	[55]
2	*Lens esculentum* Mill.	limonene	Antitumor, antiviral, anti-inflammatory, and antibacterial activities	[61, 62]
		cineole	Inflammatory	[61, 63]
	*Vitis vinifera* L	Resveratrol (3,40,5-trihydroxystilbene)	Cyclooxygenase activity	[64]
		Gallic acid	Antioxidant, anti-inflammatory, and antineoplastic properties	[63, 64]
		Catechin	Antioxidant, antibacterial, antifungal, antidiabetic, anti-inflammatory, antiproliferative and antitumor properties	[64–66]
		Proanthocyanidins	Antioxidant, antibacterial, antifungal, antidiabetic, anti-inflammatory, antiproliferative and antitumor properties	[66–68]
		Quercetin-O-pentoside	Anti-inflammatory, antioxidant activity	[65]
		Petunidin-3-O-glucoside	Antioxidant, α-glucosidase and xanthine oxidase inhibitory activities	[65]
		Rutin	Anti-bacterial, anti-inflammatory, analgesic, anti-radiation, antioxidant, anti-myocardial hypoxia activity	[52, 69]
		Caftaric acid	Antioxidant, anti-inflammatory, antimutagenic and anticarcinogenic, hepatoprotective, anti-diabetic, anti-hypertensive, anti-obesity, anti-metabolic syndrome and neuroprotective effects.	[69, 70]
	*Moringa oleifera* Lam.	Quercetin,	Anti-inflammatory, antioxidant activity	[71, 72]
		Kaempferol	Antioxidant, anti-inflammatory, antimicrobial, anticancer, cardioprotective, neuroprotective, antidiabetic, anti-osteoporotic, antiestrogenic activities	[51, 60, 72, 73]
		Ellagic acid	Antioxidant, anti-inflammatory, and antineoplastic properties	[72]
		Gallic acid	Antioxidant, anti-inflammatory, and antineoplastic properties	[64, 72]
		Ferulic acid,	Anti-inflammatory, antimicrobial, anticancer, anti-arrhythmic, and antithrombotic activity, antidiabetic effects and immunostimulant properties	[59, 72]
		Chlorogenic acid	Anti-inflammatory, antimicrobial, anticancer	[72]
		Niazimicin	Anti-inflammatory, antimicrobial, anticancer	[72]
		Rutin	Anti-bacterial, anti-inflammatory, analgesic, anti-radiation, antioxidant, anti-myocardial hypoxia activity	[52, 69]
		Moringin	Antioxidant, anti-carcinogenic, anti-diabetic, anti-inflammatory and anti-hypertensive properties	[74]
		Rutin	Anti-bacterial, anti-inflammatory, analgesic, anti-radiation, antioxidant, anti-myocardial hypoxia activity	[52, 69, 72]

On the other hand, herbal Mixture number one contains *Monsonia angustifolia* E.Mey. ex A.Rich. and *Olea europaea* L., both of which contains a planthera of pharmacologically active metabolites like matairesinol, oleuropein, hydroxytyrosol and elenoic acid with strong anti-inflammatory and anti-cancer activity. *Olea europaea* also contains rutin amongst many other compounds which as mentioned earlier has vast pharmacological activities. These and other compounds listed here could form the bases of the activities observed in the herbal mixtures.

Khorombi [76], investigated the anticancer properties of *M. angustifolia* extracts in vitro against three highly sensitive cancer cell lines, namely melanoma UACC62, renal TKIO and breast MCF7. They reported a significant inhibition of the growth of cancer cells by the organic (methanol:dichloromethane, 1:1 v/v) extract. In another study, Guo et al. [51], reported the beneficiary effect of fermenting *O. europaea* which results in conversion of oleuropein into hydroxytyrosol (HT), elenolic acid glucoside (oleoside-11-methyl ester) and oleuropein aglycone. These compounds have been shown to exhibit Antioxidant, anti-inflammatory, anti-atherogenic, anti-cancer, antimicrobial and antiviral activity [51, 52]. The fermentation of the herbal mixture with *O. europaea* could result in the production of these compounds which could contribute to the use of the mixtures.

## Conclusion

The antiviral activity of one of the herbal concoctions was higher than one of the pharmaceutical standards used for the treatment of HIV-1. Hence displaying some potential inhibition against HIV-1 RT. There was variation in the anti-inflammation activity as determined by the sPL2, 15-LOX and COX enzyme assays. The only concerning matter was the high COX-1 activity in some of the extracts, which is not desirable due to the mucosal protection action of COX-1 enzyme. The toxicological results found in this study suggest that the concoctions might likely not be toxic to human cells upon consumption except for the crude concoction 2 which appeared to show a high percentage of toxicity. Moreover, the obtained data suggested that the concoctions are selectively toxic against HT-29 cancerous cells rather than normal C3A cells. Therefore, the herbal concoctions do display valid pharmacological potential. However, more work is needed to explore more on the anti-cancer activity and mechanisms of action thereof.

## Data Availability

The datasets used and/or analyzed during the current study are available from the corresponding author on reasonable request.
